# Establishment of monoclonal anti-human CD26 antibodies suitable for immunostaining of formalin-fixed tissue

**DOI:** 10.1186/1746-1596-9-30

**Published:** 2014-02-06

**Authors:** Ryo Hatano, Taketo Yamada, Shuji Matsuoka, Satoshi Iwata, Hiroto Yamazaki, Eriko Komiya, Toshihiro Okamoto, Nam H Dang, Kei Ohnuma, Chikao Morimoto

**Affiliations:** 1 Department of Therapy Development and Innovation for Immune Disorders and Cancers, Graduate School of Medicine, Juntendo University, 2-1-1, Hongo, Bunkyo-ku, Tokyo 113-8421, Japan; 2 Department of Pathology, Keio University School of Medicine, 35 Shinanomachi, Shinjuku-ku, Tokyo 160-8582, Japan; 3 Department of Pathology & Oncology, Juntendo University School of Medicine, 2-1-1, Hongo, Bunkyo-ku, Tokyo 113-8421, Japan; 4 Division of Hematology/Oncology, University of Florida, 1600 SW Archer Road- Box 100278, Room MSB M410A, Gainesville, FL 32610, USA

**Keywords:** CD26/dipeptidyl peptidase 4, Immunohistochemical staining, Companion diagnostic drug, Malignant mesothelioma, T cell costimulation

## Abstract

**Background:**

A T cell costimulatory molecule with dipeptidyl peptidase IV (DPPIV) activity in its extracellular region, CD26 is a multifunctional molecule associated with various proteins such as adenosine deaminase, caveolin-1, CXCR4, collagen, and fibronectin, while playing an important role in the regulation of inflammatory responses and tumor biology. We have focused on CD26 as a novel therapeutic target for various tumors and immune disorders, and have developed a humanized anti-CD26 monoclonal antibody (mAb), YS110, which is currently being evaluated in a phase I clinical trial for patients with CD26-expressing tumors, including malignant mesothelioma. Since detection of tumor CD26 expression is required for determining potential eligibility for YS110 therapy, the development of anti-human CD26 mAb that can clearly and reliably detect the denatured CD26 molecule in the formalin-fixed paraffin-embedded tissues is critical.

**Methods:**

To develop novel anti-CD26 mAbs capable of binding to the denatured CD26, we immunized mice with CD26 protein denatured in urea buffer. After the fusion of splenocytes and myeloma cells, the mAbs were screened for specific reactivity with human CD26 by flow cytometry, enzyme-linked immunosorbent assay, and immunohistochemistry. The binding competitiveness of novel anti-CD26 mAbs with the humanized anti-CD26 mAb YS110 was also examined.

**Results:**

We have succeeded in developing novel anti-human CD26 mAbs suitable for immunohistochemical staining of CD26 in formalin-fixed tissue sections with reliable clarity and intensity. Importantly, some of these mAbs exhibit no cross-reactivity with the humanized anti-CD26 mAb.

**Conclusions:**

These novel mAbs are potentially useful as companion diagnostic agents to analyze CD26 expression in the clinical setting while advancing future CD26-related research.

**Virtual slides:**

The virtual slides for this article can be found here: http://www.diagnosticpathology.diagnomx.eu/vs/5987140221097729

## Introduction

CD26 is a 110-kDa type II membrane-bound glycoprotein with dipeptidyl peptidase IV (DPPIV) activity in its extra cellular domain [1-3]. CD26 is composed of 766 amino acids (AAs), and is anchored to the lipid bilayer by a single hydrophobic segment at residues 7–28. The cytoplasmic tail of CD26 is composed of only 6 amino acid residues at the N-terminal end (AA 1–6) without a common signaling motif structure. The predominant part of CD26 consists of the extra cellular domain (AA 29–766) divided into three regions, a glycosylated region, a cysteine-rich region and a C-terminal DPPIV catalytic region [4,5]. DPPIV belongs to the serine protease family, able to cleave dipeptides from polypeptides with N-terminal penultimate proline or alanine, and regulates the activities of a number of cytokines and chemokines [3]. CD26 is a multifunctional molecule associated with various proteins such as adenosine deaminase (ADA), caveolin-1, CXCR4, collagen, and fibronectin, and is expressed on various cell types, including epithelial cells (kidney proximal tubules, bile duct, prostate and intestine), endothelial cells as well as T lymphocytes [4-6]. The function of CD26 is dependent on cell types and the microenvironment, which influence its multiple biological roles [4-7].

In addition to being a marker of T cell activation, CD26 is associated with T cell signal transduction processes as a costimulatory molecule [4]. While CD26 expression is increased following activation of resting T cells, CD4^+^CD26^*high*^ T cells respond maximally to recall antigens such as tetanus toxoid [8]. Moreover, crosslinking of CD26 and CD3 with solid-phase immobilized monoclonal antibodies (mAbs) can induce T cell costimulation and IL-2 production by CD26^+^ T cells [4]. Furthermore, high CD26 cell surface expression in CD4^+^ T cells is correlated with the production of T_H_1-type cytokines and high migratory activity [4]. Taking into account the data that effector T cells in inflamed lesions express high levels of CD26, it is conceivable that CD4^+^CD26^+^ T cells play an important role in the inflammatory process [5,9,10]. We have recently found that cytotoxic activity of CD8^+^ T cells is also regulated via CD26-mediated costimulation [11]. More recently, we have shown that humanized anti-CD26 mAb appears to be a promising novel therapy for the clinical control of graft-versus-host disease (GVHD) in a xenogeneic GVHD murine model [12]. CD26 is also expressed on various tumors such as malignant mesothelioma, renal carcinoma, colon cancer, hepatocellular carcinoma, lung cancer, prostate cancer, gastrointestinal stromal tumor (GIST), thyroid cancer, T-lymphoblastic lymphoma and T-acute lymphoblastic leukemia [13]. We have shown that administration of anti-CD26 mAb resulted in both in vitro and in vivo inhibition of tumor cell growth, migration and invasion, and enhanced survival of mouse xenograft models inoculated with T-lymphoma, renal cell carcinoma or malignant mesothelioma [14-16]. Based on these findings, we have focused on CD26 as a novel therapeutic target for various tumors and immune disorders, and have developed a humanized anti-CD26 mAb, YS110, which is being investigated currently in a phase I clinical trial for patients with CD26-expressing tumors, including malignant mesothelioma [17].

The development of companion diagnostic agents to be used in conjunction with the appropriate therapeutic modalities is essential to maximize therapeutic effectiveness while minimizing associated toxicities. Detection of tumor CD26 expression is critical to determining potential eligibility for treatment with humanized anti-CD26 mAb, and it is also important to determine whether CD26 expression on tumors or lymphocytes is affected by anti-CD26 mAb therapy. Immunohistochemical staining of CD26 with the many anti-CD26 mAbs previously developed in our laboratory [18] did not reveal an anti-CD26 mAb that can clearly detect the denatured CD26 molecule in formalin-fixed paraffin-embedded tissues. Meanwhile, testing of several commercially available anti-CD26 mAbs designated as research reagents for immunohistochemical staining, and a mAb purchased from MBL indicated that these mAbs could stain the denatured CD26 in formalin-fixed tissues, but not with sufficient clarity. On the other hand, our testing of commercially available anti-CD26 polyclonal antibodies (pAbs), and a pAb purchased from R&D Systems showed that these reagents exhibited reliable staining pattern and intensity [19]. However, the disadvantage of pAbs is the potential lot-to-lot variability in staining pattern and intensity, which makes pAbs not the ideal reagents for diagnostic testing of patient specimens in the clinical setting, where consistency and uniformity are required.

In the present study, by immunizing mice with CD26 protein denatured in urea buffer, we have succeeded in developing novel anti-human CD26 mAbs that can be used as companion diagnostic reagents suitable for immunohistochemical staining of CD26 in formalin-fixed tissue sections with reliable clarity and intensity. In addition, since some of these mAbs display no cross-reactivity with the therapeutic humanized anti-CD26 mAb, they may be suitable for assays analyzing CD26 expression during or following treatment with the humanized anti-CD26 mAb.

## Materials and methods

### Animals

Female BALB/c mice were purchased from CLEA Japan (Tokyo, Japan) and housed in a specific pathogen-free facility in micro-isolator cages. Animal experiments were conducted following protocols approved by the Animal Care and Use Committee at Juntendo University.

### Antibodies

To determine the epitope of the newly developed mouse anti-human CD26 mAbs, murine anti-human CD26 mAbs (clone 4G8, 1F7, 14D10, 5F8, 16D4B or 9C11) which have been already developed in our laboratory were used [18]. We have previously shown that these mAbs are divided into 5 separate groups by their epitopes, 4G8 recognizing the 1-247th AAs region of CD26, 1F7 and 14D10 recognizing the 248-358th AAs region of CD26, 5F8 recognizing the 359-449th (closer to the 359th) AAs region of CD26, 16D4B recognizing the 450-577th AAs region of CD26, and 9C11 recognizing the 359-653th (but different from 5F8 or 16D4B) AAs region of CD26. The humanized anti-CD26 mAb (YS110) was generated by utilizing the complementarity determining regions of the murine anti-human CD26 mAb 14D10 [18], and generously provided by Y's Therapeutics (Tokyo, Japan). To compare the staining pattern and intensity of human CD26 on formalin-fixed tissue sections, we used two commercial anti-human CD26 Abs available for CD26 detection by immunohistochemistry. One is the culture supernatant form of a mouse anti-human CD26 mAb (clone 44–4) purchased from MBL (Nagoya, Japan), and the other is a purified goat anti-human CD26 pAb purchased from R&D Systems (Minneapolis, MN). Human polyclonal IgG (venilon-I) was purchased from Alfresa Corporation (Tokyo, Japan), and mouse IgG_1_ isotype control (clone MG1-45) was purchased from BioLegend (San Diego, CA). YS110, control human IgG, 4G8, 1F7, 5F8, 16D4B, 9C11, purified clone 18, clone 19, and mouse IgG_1_ isotype control were labeled using an Alexa Fluor 647 Monoclonal Antibody Labeling Kit (Molecular Probes, Eugene, OR) according to the manufacturer's instructions.

### cDNA constructs and transfection

As described previously [18], C-terminal deletion mutants of human CD26 cDNA constructs were generated by using Nco I restriction enzyme sites to delete domain representing the 740-766th AAs in the C terminus, using Nhe I to delete from the 578th AA, using BspE I to delete from the 450th AA, using Stu I to delete from the 359th AA, and using Pst I to delete from the 248th AA. These cDNAs were ligated in-frame into pcDL-SRα expression vector [20]. The green fluorescence protein (GFP)-expressing vector pEB6-CAG-GFP was a kind gift from Dr. Yoshihiro Miwa (Tsukuba University, Tsukuba, Japan) [21]. Each CD26 deletion construct in pcDL-SRα was co-transfected with pEB6-CAG-GFP into COS-7 cells using Lipofectamine 2000 reagent (Invitrogen, Carlsbad, CA). After 24 hours of transfection, cells were harvested, followed by staining with Alexa Fluor 647-labeled 4G8, 1F7, 5F8, YS110, clone 18 or clone 19, and then analyzed by flow cytometry.

### Preparation of immunogen

Soluble CD26 (sCD26) was produced according to the method described previously [22]. Briefly, the expression vector RcSRα-26d3-9, which contains a deletion of the coding sequence for amino acids 3–9 of CD26, was transfected into a dihydrofolate reductase deficit Chinese hamster ovary (CHO) cell line, DXB-11 by electroporation, together with pMT-2 providing the dihydrofolate reductase gene. The transfected CHO cells were cultured in serum-free CHO-S-SFM II medium (Invitrogen) supplemented with 1 μM methotrexate (Nacalai Tesque, Kyoto, Japan). The culture supernatant was collected and subjected to affinity chromatography on ADA-Sepharose according to the method described previously [23]. Purified sCD26 was denatured in 8 M urea buffer supplemented with 20 mM HEPES and 50 mM dithiothreitol (DTT) by gentle rotation for 8 hours at RT.

### Development of hybridomas and monoclonal anti-human CD26 antibodies

Denatured sCD26 was dialyzed in PBS, and 100 μg of protein per 50 μl of PBS was emulsified with 50 μl of adjuvant, TiterMax Gold (TiterMax USA, Norcross, GA). A 6-wk-old female BALB/c mouse was immunized s.c. with 100 μl of the emulsion seven times every two weeks and finally injected i.v. with half volume of the emulsion. Three days after the final immunization, the spleen was removed and 100 × 10^6^ spleen cells were fused with 100 × 10^6^ P3U1 myeloma cells by using polyethylene glycol 4000 (Merck, Darmstadt, Germany) and were cultured in RPMI1640 supplemented with 10% fetal bovine serum (FBS, Japan Bioserum, Fukuyama, Japan), 5% BriClone (NICB, Dublin, Ireland) and HAT (Invitrogen) in 96-well flat-bottom plates (Costar, Corning Incorporated, Corning, NY). Hybridoma supernatants were screened for selective reactivity with human CD26 by using flow cytometry and enzyme-linked immunosorbent assay (ELISA). The supernatants which can detect human CD26 by both flow cytometry and ELISA were finally screened for immunostaining of formalin-fixed paraffin-embedded human tissue sections. The hybridomas were cloned by limiting dilution and culture medium was exchanged for serum-free GIT medium (Wako Pure Chemicals, Osaka, Japan). Monoclonal antibodies were purified from the supernatants using Protein A IgG Purification Kit (Pierce, Rockford, IL) according to the manufacturer’s instructions.

### Flow cytometry

A CD26-negative Jurkat T cell line (Jurkat parent) and a stable Jurkat T cell line transfected with human CD26 cDNA (Jurkat-CD26WT) described previously [24] were used for screening of hybridomas. Cells were washed in PBS containing 1% FBS, 1 mM EDTA and 0.1% sodium azide, and incubated with 100 μl of hybridoma supernatant or 20 μg/ml of purified mouse anti-human CD26 mAb for 25 min at 4°C, and subsequently stained with PE-conjugated goat anti-mouse Ig pAb (BD Biosciences, San Jose, CA) for 25 min at 4°C. Acquisition was performed using FACSCalibur (BD Biosciences) and data were analyzed with FlowJo software (Tree Star, Ashland, OR). For cross-blocking studies of humanized anti-CD26 mAb (YS110), cells were pretreated with unlabeled YS110 or control human IgG (50 μg/ml, respectively) for 25 min at 4°C, and subsequently incubated with 100 μl of hybridoma supernatant or 20 μg/ml of purified mouse anti-human CD26 mAb for 25 min at 4°C, and finally stained with PE-conjugated goat anti-mouse Ig pAb for 25 min at 4°C. For cross-blocking studies of murine anti-CD26 mAbs, cells were pretreated with unlabeled 4G8, 1F7, 5F8, 16D4B, 9C11 or mouse IgG_1_ isotype control (50 μg/ml, respectively) for 25 min at 4°C, and subsequently stained with Alexa Fluor 647-labeled clone 18 or clone 19 or PE-conjugated goat anti-mouse Ig pAb for 25 min at 4°C.

### ELISA

The 96-well immunoplates (NUNC, Roskilde, Denmark) were coated with native sCD26 or denatured sCD26 described above in carbonate bicarbonate buffer (200 ng/well, respectively) or buffer alone as a negative control at 4°C overnight. Each well of the plate was blocked with 3% bovine serum albumin (BSA, Sigma, St.Louis, MO) in PBS for 1 hour at RT, and then incubated with 3-fold diluted hybridoma supernatants or 5 μg/ml of purified mouse anti-human CD26 mAb or goat anti-human CD26 pAb for 1 hour at RT, and subsequently incubated with horseradish peroxidase (HRP)-conjugated goat anti-mouse Ig pAb (BD Biosciences) or HRP-conjugated donkey anti-goat IgG Ab (Santa Cruz Biotechnology, Santa Cruz, CA) for 1 hour at RT. Tetramethylbenzidine (TMB) Peroxidase Substrate (KPL, Gaithersburg, MD) was finally added to each well and the reaction was stopped by 2N H_2_SO_4_. The absorbance at 450 nm/570 nm was measured in a Microplate Reader (Bio-Rad, Hercules, CA) and data were analyzed with Microplate Manager 6 software (Bio-Rad).

### Tissue specimens and immunohistochemical staining

Formalin-fixed paraffin-embedded tissue specimens of malignant mesothelioma and normal liver, kidney and prostate were used for positive controls in the immunohistochemical examination. The use of human sample from autopsy cases with hepatocellular carcinoma, renal cell carcinoma, prostate adenocarcinoma, colon adenocarcinoma and lung adenocarcinoma was generously permitted by the bereaved families. This study was approved by the Okayama Rosai Hospital ethical review board and the Keio University School of Medicine ethical review board, and the purpose of the study was explained to all patients and their written informed consent was obtained. All studies on human subjects were carried out according to the principles set out in the Declaration of Helsinki. Formalin-fixed paraffin-embedded tissue specimens were cut into 4-6 μm sections and deparaffinized. Antigen retrieval was performed by 1) autoclaving in 10 mM citrate buffer (pH 6.0) for 20 min at 120°C, 2) 0.05% trypsin for 15 min at 37°C, 3) 0.02% proteinase K for 10 min at 37°C, or 4) boiling in 10 mM citrate buffer (pH 6.0) for 10 min at 100°C, and the sections were treated with 0.3% H_2_O_2_ in methanol for 10 min at RT to inactivate endogenous peroxidase, then treated with 2.5% horse serum (Vector Laboratories, Burlingame, CA) for 10 min at RT to block non-specific binding of the secondary horse antibody. The sections were treated with 100 μl of hybridoma supernatants or purified mouse anti-human CD26 mAb or goat anti-human CD26 pAb for 2 hours at RT, and subsequently treated with HRP-conjugated horse anti-mouse Ig pAb or HRP-conjugated horse anti-goat IgG pAb (Vector Laboratories) for 30 min at RT. The reaction was visualized with 3, 3’-diaminobenzidine (DAB) (Dojindo Laboratories, Kumamoto, Japan), and the tissue sections were counterstained for nucleus with hematoxylin. To confirm the binding specificity of Abs to human CD26, the anti-human CD26 Ab (100 μg/ml) was gently rotated with 500 μg/ml of sCD26 at 4°C overnight, and after centrifugation, the supernatant was used instead of the primary anti-human CD26 Ab. Expression pattern of CD26 was evaluated and verified independently by two pathologists. The optical microscope images were taken using Axio Scope.A1 microscope (Carl Zeiss, Oberkochen, Germany).

## Results

### Screening of hybridoma cells

To develop a novel anti-CD26 mAb capable of binding to the denatured CD26, we immunized mice with CD26 protein denatured in urea buffer. To determine the denaturing condition, we incubated CD26 protein in 8 M urea buffer at RT for 30 min, 3 hours or 12 hours, and analyzed the binding of anti-CD26 mAb (clone 5F8) or anti-CD26 pAb (R&D Systems) to the urea treated CD26 protein by ELISA as described in Materials and Methods. This analysis showed the decrease in the absorbance when CD26 protein was incubated for 30 min in urea buffer, with additional decrease in absorbance at 3 hours of incubation, while there was barely noticeable difference between 3 hours of incubation and 12 hours of incubation (data not shown). These data suggest that most of the CD26 proteins were denatured when incubated in 8 M urea buffer for more than 3 hours, and we used this urea-treated CD26 protein as an immunogen.

**Figure 1:**
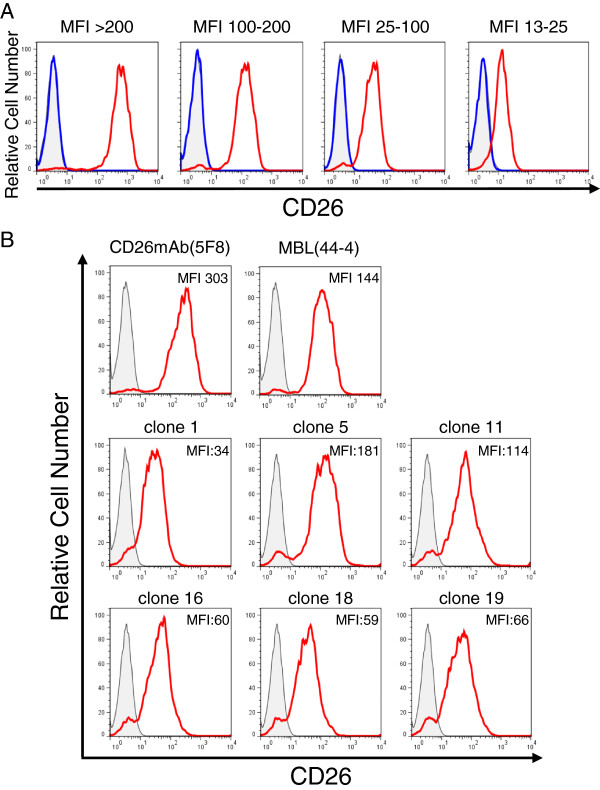
**Flow cytometry analysis with novel anti-CD26 mAbs. A.** Jurkat-CD26WT cells (red lines) or Jurkat parent cells (blue lines) were incubated with the hybridoma supernatant, and subsequently stained with PE-labeled anti-mouse Ig pAb, and analyzed by flow cytometry. **B.** Jurkat-CD26WT cells were incubated with the hybridoma supernatant (clone 1, 5, 11, 16, 18 or 19) or purified mouse anti-CD26 mAb (5F8) or commercial mouse anti-CD26 mAb (MBL, clone 44–4), and subsequently stained with PE-labeled anti-mouse Ig pAb, and analyzed by flow cytometry. The gray areas in each histogram show the data involving the isotype control. The mean fluorescence intensity (MFI) of each staining is shown. Data shown are repeated twice **(A)** and five times **(B)** with similar results.

After the fusion of splenocytes and P3U1 myeloma cells, the culture supernatant was collected and screened for selective reactivity with human CD26. For the first screening of hybridoma cells, we used an endogenous CD26-deficit Jurkat cell line (Jurkat parent) and a stable Jurkat cell line transfected with full-length human CD26 (Jurkat-CD26WT), and the binding to human CD26 was analyzed by flow cytometry. As shown in Figure 1A, we obtained a number of hybridomas secreting antibodies, some of which could stain Jurkat-CD26WT with bright intensity and others could stain with intermediate or dull intensity (red lines) while Jurkat parent cells showed no staining with all of these supernatants (blue lines). These data indicate that this screening method excludes the possibility of non-specific binding to other proteins beside CD26. The representative histograms of these novel anti-CD26 mAbs available for immunohistochemical staining were shown in Figure 1B.

**Figure 2:**
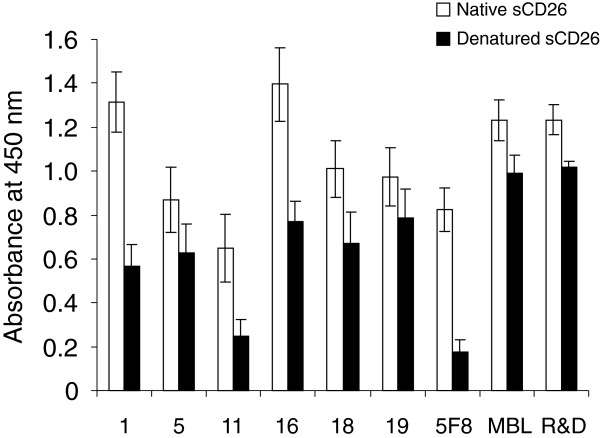
**ELISA analysis with novel anti-CD26 mAbs.** Non treated native soluble CD26 (sCD26) or urea treated denatured sCD26 was incubated with the hybridoma supernatant (clone 1, 5, 11, 16, 18 or 19) or purified mouse anti-CD26 mAb (5F8) or commercial mouse anti-CD26 mAb (MBL, clone 44–4) or purified goat anti-CD26 pAb (R&D Systems). The absorbance at 450 nm/570 nm was measured, and data are shown as mean ± S.E. from three independent experiments.

The positive supernatants were then screened by ELISA for reactivity with native or denatured (urea treated) sCD26 protein. To exclude the possibility of non-specific binding to BSA used for blocking, we prepared the wells coated with buffer alone (without sCD26), subsequently blocked with BSA and incubated with hybridoma supernatants. The absorbance of the wells at 450 nm was subtracted from the absorbance of the wells coated with native or denatured sCD26. The clone was judged to be positive if the absorbance to the native sCD26 was higher than 0.1. The absorbance to the native or denatured sCD26 was quite different from clone to clone, and the representative absorbance of novel anti-CD26 mAbs available for immunohistochemical staining was shown in Figure 2. When sCD26 was denatured in urea buffer, the absorbance of 5F8, which cannot detect denatured CD26 in formalin-fixed tissues, was apparently decreased, while the absorbance of commercial mAb (purchased from MBL) or pAb (purchased from R&D Systems) was comparatively maintained (Figure 2). Although the decrease of absorbance to the denatured sCD26 was also observed with the novel anti-CD26 mAbs, particularly with clone 1, clone 11 and clone 16, the absolute value of absorbance to the denatured sCD26 was much higher than that of 5F8, except for clone 11 (Figure 2). As a result of the screening, 31 clones that secreted anti-human CD26 mAbs were evaluated for both flow cytometry and ELISA.

### Immunohistochemical staining with novel anti-CD26 mAbs

**Figure 3:**
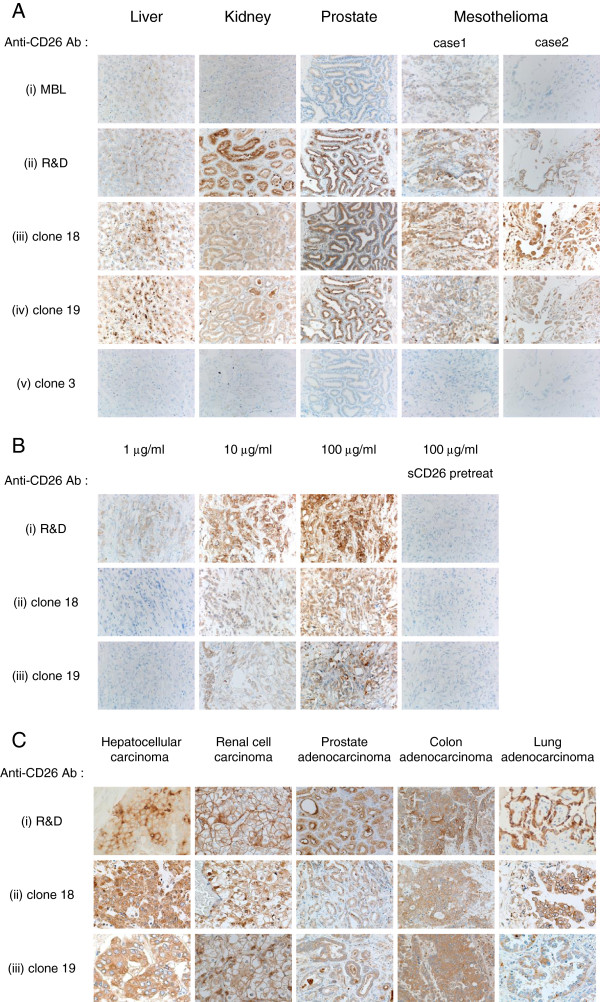
**Representative results of immunostaining with novel anti-CD26 mAbs. A.** The tissue specimens of liver, kidney, prostate or two cases of malignant mesothelioma were stained with 100 μl of commercial mouse anti-human CD26 mAb supernatant (MBL, clone 44–4) (i), or 10 μg/ml of purified goat anti-human CD26 pAb (R&D Systems) (ii), or newly developed hybridoma supernatant (clone 18 (iii), clone 19 (iv) or clone 3 (v)). **B**. Malignant mesothelioma tissue specimens were stained with commercial goat anti-human CD26 pAb (R&D Systems) (i), or purified novel mouse anti-human CD26 mAbs (clone 18 (ii) or clone 19 (iii)) at the indicated concentrations of Abs in the presence or absence of sCD26. **C.** The tissue specimens of hepatocellular carcinoma, renal cell carcinoma, prostate adenocarcinoma, colon adenocarcinoma or lung adenocarcinoma were stained with 100 μg/ml of commercial goat anti-human CD26 pAb (R&D Systems) (i), or purified mouse anti-human CD26 mAbs (clone 18 (ii) or clone 19 (iii)). All specimens were counterstained with hematoxylin (original magnification, 200X).

To determine whether the newly developed anti-CD26 mAbs were suitable for immunohistochemical staining of CD26 in formalin-fixed tissue sections, surgically resected tissue specimens of normal liver, kidney, prostate, and malignant mesothelioma were immunostained with these mAbs, with commercial anti-CD26 mAb (purchased from MBL) and anti-CD26 pAb (purchased from R&D Systems) being used as controls. Although we examined several antigen retrieval conditions, tissue specimens stained with anti-CD26 mAb purchased from MBL exhibited only a slightly positive reaction with weak staining intensity, revealing this mAb to be inappropriate for the detection of CD26 expression in formalin-fixed clinical samples (Figure 3A-i). In contrast, tissue specimens stained with anti-CD26 pAb purchased from R&D Systems exhibited a clear staining pattern of CD26, namely the surface membrane of bile canaliculi, the brush border of renal proximal tubular epithelial cells and prostate epithelial cells were specifically stained with low background (Figure 3A-ii). We have previously shown that CD26 was also highly expressed in various pathologic types of malignant mesothelioma, including localized malignant mesothelioma, well-differentiated papillary malignant mesothelioma, and diffuse malignant mesothelioma [16], and the specific staining of malignant meshothelioma cells was also observed with the use of the anti-CD26 pAb (Figure 3A-ii). After testing the hybridoma supernatants from the 31 clones described above for immunohistochemical staining, we finally obtained 6 clones (clone 1, 5, 11, 16, 18 or 19) capable of staining CD26 in formalin-fixed tissues with much stronger intensity than the mAb purchased from MBL. As shown in Figure 3A, tissue specimens stained with two representative clones (clone 18 or 19) exhibited reliable staining pattern and intensity comparable to the pAb purchased from R&D Systems (panels iii and iv), while no apparent staining of CD26 was observed in the specimens stained with clone 3 (judged to be negative for immunostaining) (panel v). Representative results of immunostaining with the other 4 clones (clone 1, 5, 11 or 16) were shown in Additional file 1: Figure S1.

We next examined immunohistochemical staining with purified novel mAbs instead of the hybridoma culture supernatants. To determine the optimal Ab concentration for immunostaining, we evaluated the anti-human CD26 Abs in concentrations ranging from 1 μg/ml to 100 μg/ml. As shown in Figure 3B, staining of malignant mesothelioma cells was hardly observed with 1 μg/ml of clone 18, clone 19 mAb or pAb purchased from R&D Systems, while the staining intensity was enhanced in a dose-dependent manner up to 100 μg/ml of these three Abs (panels i, ii, iii). Meanwhile, staining of tissues with even higher Ab concentrations resulted in similar intensity as compared with those stained with 100 μg/ml of the Abs (data not shown). In addition, to confirm the binding specificity of these Abs to human CD26, the sections were treated with purified anti-human CD26 Ab preincubated with sCD26. As shown in Figure 3B, the binding of these Abs was completely inhibited by sCD26 (panels i, ii, iii). These results indicate that the newly developed mAbs specifically bind to human CD26, and 100 μg/ml seems to be an optimal concentration of these Abs for immunohistochemical staining.

We further examined immunohistochemical staining of CD26-expressing tumor tissues other than malignant mesothelioma (hepatocellular carcinoma, renal cell carcinoma, prostate adenocarcinoma, colon adenocarcinoma, and lung adenocarcinoma) with the purified mAb of clone 18 or 19. As shown in Figure 3C, each tumor tissue stained with clone 18 or 19 (panels ii and iii) exhibited clarity and intensity similar to the levels observed with the anti-CD26 pAb purchased from R&D Systems (panel i). Results from the immunostaining studies indicate that CD26 can be detected both on the cell surface as well as cytoplasm of these carcinoma tissues.

### Cross-blocking studies with humanized anti-CD26 mAb

**Figure 4:**
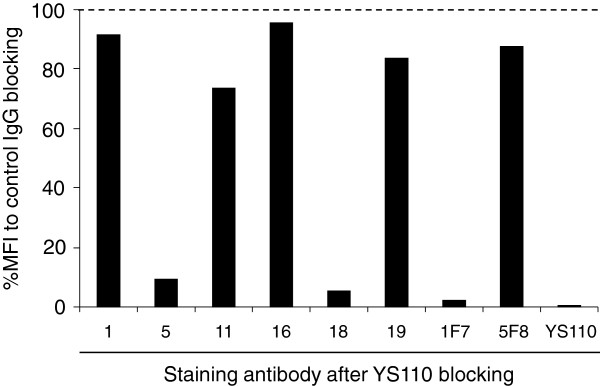
**Analysis of crossreactivity of novel anti-CD26 mAbs with humanized anti-CD26 mAb.** Jurkat-CD26WT cells were pretreated with unlabeled humanized anti-CD26 mAb (YS110) or human control IgG, and then treated with the hybridoma supernatant (clone 1, 5, 11, 16, 18 or 19) or purified mouse anti-CD26 mAb (1F7 or 5F8), and subsequently stained with PE-labeled anti-mouse Ig pAb. For staining with humanized anti-CD26 mAb, cells were stained with Alexa Fluor 647-labeled YS110 after pretreatment with unlabeled YS110. Data were analyzed by flow cytometry, and the percentage of mean fluorescence intensity (MFI) after YS110 blocking to MFI after control IgG blocking is shown. Data shown are repeated twice with similar results.

In addition to detecting CD26 expression on tumor cells or lymphocytes prior to the therapeutic administration of humanized anti-CD26 mAb, it is also important to evaluate whether anti-CD26 mAb therapy affects CD26 expression on relevant tissues. For this purpose, we next examined the binding competitiveness of the 6 novel anti-CD26 mAbs with the humanized anti-CD26 mAb YS110. Jurkat-CD26WT was pretreated with unlabeled YS110 or control human IgG for 25 min, subsequently incubated with hybridoma supernatants, and stained with PE-labeled anti-mouse Ig pAb. As shown in Figure 4 (representative histograms are shown in Additional file 1: Figure S2), binding of YS110 or 1F7 to CD26 was completely blocked by YS110 while the binding of 5F8 to CD26 was hardly affected, indicating that YS110 was sufficiently bound to CD26. Although binding of clone 1, 11, 16 or 19 to CD26 was hardly affected by YS110 pretreatment, binding of clone 5 was partially inhibited, and binding of clone 18 was completely inhibited by YS110 (Figure 4 and Additional file 1: Figure S2). Taken together, these data suggest that clone 19 was capable of detecting denatured CD26 in formalin-fixed tissue sections with the most reliable staining pattern and intensity, exhibited no cross-reactivity with YS110, and was suitable for analysis of CD26 expression on clinical samples following the administration of YS110.

### Epitope mapping of novel anti-CD26 mAbs

**Figure 5:**
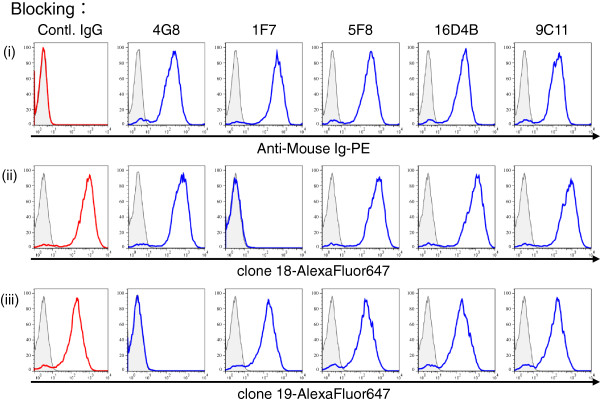
**Blocking experiment of novel anti-CD26 mAb binding to CD26.** Jurkat-CD26WT cells were pretreated with unlabeled mouse anti-CD26 mAbs (4G8, 1F7, 5F8, 16D4B, or 9C11) (blue lines) or mouse IgG_1_ isotype control (Contl. IgG) (red lines), and subsequently stained with PE-labeled anti-mouse Ig pAb **(i)** or Alexa Fluor 647-labeled anti-CD26 mAbs (clone 18 **(ii)** or clone 19 **(iii)**), and analyzed by flow cytometry. The representative histograms of CD26 expression are shown, and the gray areas in each histogram show the data involving the isotype control. Data shown are repeated twice with similar results.

To define the CD26 epitope recognized by clone 18 and 19, we conducted cross-blocking studies using anti-CD26 mAbs with epitopes that had been extensively characterized previously as described in Materials and Methods [18]. To confirm the binding of anti-CD26 mAbs to CD26, Jurkat-CD26WT was incubated with unlabeled 4G8, 1F7, 5F8, 16D4B, 9C11 or mouse IgG_1_ isotype control for 25 min, and subsequently stained with PE-labeled anti-mouse Ig pAb. As shown in Figure 5-i, each anti-CD26 mAb was sufficiently bound to CD26 (blue lines), while there was no binding of the isotype control (red line). Modulation of cell surface CD26 into the cytoplasm following treatment with these anti-CD26 mAbs did not occur under these experimental conditions. Similarly, Jurkat-CD26WT was pretreated with unlabeled anti-CD26 mAbs, and subsequently stained with Alexa Fluor 647-labeled clone 18 or 19. As shown in Figure 5, binding of clone 18 or 19 to CD26 was completely inhibited by 1F7 (panels ii) or 4G8 (panels iii), respectively, with no effect by the other anti-CD26 mAbs. These results suggest that the epitope defined by clone 18 might be identical to 1F7, locating between the 248-358th AAs region of CD26, while the epitope defined by clone 19 might be identical to 4G8, locating between the 1-247th AAs region of CD26.

For cross-blocking studies involving the other 4 novel anti-CD26 mAbs, Jurkat-CD26WT was incubated with unlabeled clone 1, 5, 11, 16, 18, 19 or mouse IgG_1_ isotype control for 25 min, and subsequently stained with Alexa Fluor 647-labeled 4G8, YS110, 5F8, 16D4B or 9C11. As shown in Additional file 1: Figure S3, binding of 4G8 to CD26 was completely blocked by clone 19, and binding of YS110 to CD26 was completely inhibited by clone 18 and partially inhibited by clone 5, consistent with the results shown in Figures 4 and 5. Clone 1 blocked completely the binding of 9C11 and partially the binding of 16D4B to CD26, while clone 16 completely inhibited the binding of both 9C11 and 16D4B to CD26 (Additional file 1: Figure S3). On the other hand, clone 11 inhibited the binding of 5F8 to CD26 completely (Additional file 1: Figure S3). These results strongly suggest that the novel anti-CD26 mAbs have a wide range of epitopes and can be broadly divided into 4 separate groups; the epitope of clone 19 being similar to 4G8, the epitopes of clone 5 and 18 being similar to 1F7 and YS110, the epitope of clone 11 being similar to 5F8, and the epitopes of clone 1 and 16 being similar to 9C11 (clone 16 is also similar to 16D4B).

**Figure 6:**
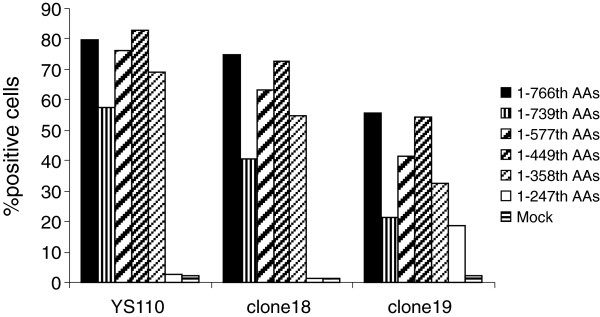
**Staining for CD26 expression on COS-7 cells transfected with CD26 deletion mutants by novel anti-CD26 mAbs.** cDNA of deleted CD26 was cotransfected with GFP-expressing plasmid to COS-7 cells. After 24 h, the transfected cells were stained with Alexa Fluor 647-labeled anti-CD26 mAbs (YS110, clone 18 or clone 19) or isotype control, and analyzed by flow cytometry. Following gating for GFP positive cells among all acquired cells, the percentage of CD26 positive cells was analyzed. Data shown are repeated twice with similar results.

To further confirm the epitope involved in binding of clone 18 and 19 to human CD26, we tested the ability of these two mAbs to bind to CD26 deletion mutants by flow cytometry [18]. We first tested the binding of the previously developed anti-human CD26 mAbs, 4G8, 1F7, or 5F8 to confirm the expression pattern of CD26 deletion mutants on COS-7 cells. As shown in Additional file 1: Figure S4, 4G8 recognized full-length CD26 and all 5 CD26 deletion mutants while 1F7 or 5F8 lost the ability to recognize the CD26 molecule with deletion from the 248th AA or from the 359th AA, respectively, indicating that the expression patterns of CD26 deletion mutants were identical to those reported previously [18]. We then analyzed the binding of YS110, clone 18 and clone 19 to the CD26 deletion mutants. As shown in Figure 6 (representative histograms are shown in Additional file 1: Figure S4), both YS110 and clone 18 recognized full length CD26, the 1-739th AAs region of CD26, the 1-577th AAs region of CD26, the 1-449th AAs region of CD26 and the 1-358th AAs region of CD26, but lost the ability to recognize the CD26 molecule with deletion from the 248th AA, suggesting that the sequence of the 248-358th AAs region on CD26 might be important for binding of YS110 and clone 18. On the other hand, clone 19 recognized full-length CD26 and all 5 CD26 deletion mutants, suggesting that the epitope defined by clone 19 might be located between the 1-247th AA region (Figure 6 and Additional file 1: Figure S4). YS110, clone 18 and clone 19 did not bind to COS-7 cells transfected with vector alone (mock) (Figure 6 and Additional file 1: Figure S4). Taken together, results from the cross-blocking studies and those involving CD26 deletion mutants strongly suggest that the epitope defined by clone 19 may be located between the 1-247th AAs region, and the epitope defined by clone 18 between the 248-358th AAs region, being almost identical to YS110.

## Discussion

Although anti-human CD26 mAbs which we have developed previously or commercially available mAbs cannot clearly detect denatured CD26 in formalin-fixed paraffin-embedded tissues, the anti-human CD26 pAb purchased from R&D Systems is able to stain CD26 with reliable clarity and intensity. However, it is of concern that the staining pattern and intensity may differ among different lots of the anti-CD26 pAb. Since treatment with targeted therapeutic agents depends on the ability to reliably detect the appropriate targets on clinical samples, uniformity of the diagnostic reagents is critical, suggesting that pAbs that are used as research reagents are not appropriate for diagnostic uses in the clinical setting. In the present study, we describe the successful development of novel anti-human CD26 mAbs by immunizing mice with CD26 protein denatured in urea buffer that can potentially be used as diagnostic reagents clinically.

In an attempt to improve diagnostic accuracy, markers used for immunohistochemistry have been studied, such as galectin-3, HBME-1 and CK-19 for diagnosis of benign and malignant thyroid lesions [25,26], and FAP-α and Calponin for diagnosing whether ductal carcinoma in situ has microinvasion [27]. CD26 is highly expressed on the surface of malignant mesothelioma cells especially tumors of the epitheloid and biphasic types, but not on benign mesothelial tissues [16,17]. It has been recently reported that the expression level of CD26 in prostate cancer tissues is higher than that of normal prostatic tissues and increased with prostate cancer stage advancement, and CD26 expression is correlated with prostate specific antigen, suggesting that CD26 may be a good marker for prostate cancer diagnosis [28]. Furthermore, the overall survival of patients with CD26-positive GISTs is worse than that of patients with CD26-negative GISTs, suggesting that CD26 appears to be a reliable biomarker of malignant GIST of the stomach [29]. These observations strongly suggest that immunohistochemical staining of CD26 in formalin-fixed tumor tissues is important for diagnosis and prognosis of various tumors.

Since several anti-human CD26 mAbs such as Ta1, 1F7, 5F8 and 14D10 that were already developed in our laboratory by immunizing mice with a CD26 positive human T cell line (EL156) or a PHA-stimulated *Aotus trivirgatus* T cell line or a murine pre-B human CD26 transfectant (300–19) cannot clearly detect CD26 in formalin-fixed tissues [1,8,18], it was our hypothesis that utilizing human CD26 protein but not human CD26 positive cells as an immunogen would be important for the development of mAbs capable of recognizing the denatured CD26 molecule. Similar to CD26, only pAbs could react to the denatured HLA class I molecules in formalin-fixed paraffin-embedded tissues. Torigoe et al. recently succeeded in developing a novel anti-pan HLA class I mAb suitable for immunohistochemical staining of fixed tissues by immunizing a recombinant HLA-A protein denatured in urea buffer [30]. The exact role played by urea treatment of the CD26 protein in expanding the repertoire of the obtained anti-CD26 mAs is not yet clear, since we have not examined for potential differences in the characteristics of mAbs obtained after immunizing mice with urea-treated sCD26 protein or non-treated native sCD26 protein in this study. However, as shown in Figures 2 and 3, tissue specimens stained with anti-CD26 mAb purchased from MBL exhibited only a partially positive reaction with weak staining intensity, while this mAb showed higher absorbance to the urea treated sCD26 protein than the absorbance obtained from the novel anti-CD26 mAbs capable of staining CD26 with strong intensity in fixed tissues. These data strongly suggest that the structure of CD26 denatured by the method of antigen retrieval after formalin-fixation is quite different from that of CD26 denatured by urea buffer, and also suggest that anti-CD26 mAbs suitable for immunohistochemistry may be obtained more efficiently by immunizing mice with CD26 protein denatured by methods other than urea treatment, such as treatment with guanidine hydrochloride or sodium dodecyl sulfate (SDS), or with proteases such as trypsin or proteinase K, or by boiling. Further studies are needed to clarify the issue involving pretreatment of the immunogens and the characteristics of mAbs obtained after immunization.

In the present study, we have succeeded in developing novel anti-CD26 mAbs with a wide range of epitopes (Figures 5, 6 and Additional file 1: Figure S3). Since most of these novel mAbs completely inhibited the binding of the anti-CD26 mAbs (4G8, 1F7, 5F8, 16D4B or 9C11) developed previously by our group, the epitopes defined by these novel mAbs are expected to be similar to those recognized by the earlier mAbs. However, these novel anti-CD26 mAbs are capable of detecting denatured CD26 in fixed tissues with strong intensity, unlike the previously developed mAbs. Similarly, while clone 18 and YS110 recognize the similar epitope on CD26 (Figure 4, Additional file 1: Figure S2 and Additional file 1: Figure S3), only clone 18 can stain CD26 clearly in fixed tissues with strong intensity, suggesting that slight differences in the recognized epitopes can determine whether mAb binding to its denatured antigen can occur.

Cross-blocking studies showed that, in contrast to clone 5 or 18, the binding of clone 1, 11, 16 or 19 to CD26 was hardly affected by the humanized anti-CD26 mAb YS110 (Figure 4, Additional file 1: Figure S2 and Additional file 1: Figure S3), suggesting that these 4 novel anti-CD26 mAbs are suitable for analyzing CD26 expression in clinical samples following YS110 therapy. Potential uses of these novel mAbs in the clinical setting may be to detect CD26 expression in formalin-fixed tissues, or on circulating cells in blood samples, or sCD26 levels in bodily fluids through such methods as immunohistochemistry, flow cytometry, or ELISA. Furthermore, these novel mAbs are potentially useful for analyzing CD26 expression in fixed tissues or on the surface of lymphocytes or tumors during or following the administration of humanized anti-CD26 mAb in animal disease models that involve inoculated human lymphocytes or tumors [12,16], and are expected to contribute to future CD26-related research effort.

Since we intend to utilize these novel anti-CD26 mAbs as companion diagnostic agents in the clinical setting, our current effort is focused on improving immunohistochemical staining methods by examining such issues as the condition of antigen retrieval or blocking, or the optimal concentration of the primary antibody (anti-CD26 mAb) that can maximize staining intensity while lowering background staining. Furthermore, we also identified the amino acid sequence of the variable region in both the heavy chain and light chain of clone 19 (data not shown), and will aim to refine the ability of this mAb to bind to CD26 through genetic engineering techniques.

In conclusion, we have succeeded in developing novel anti-human CD26 mAbs suitable for immunohistochemical staining of CD26 in formalin-fixed tissue sections with reliable clarity and intensity. Furthermore, since some of these mAbs exhibit no cross-reactivity with the therapeutic humanized anti-CD26 mAb, they are potentially useful as companion diagnostic agents in the clinical setting while advancing future CD26-related research.

## Competing interests

The authors declare no competing financial interests associated with this manuscript.

## Authors’ contributions

RH and CM designed and coordinated the study. RH, TY, SM and EK conducted the experiments. CM, TY and KO supervised part of the experiments. All authors contributed to the interpretations and conclusions presented. RH and CM wrote the manuscript, and NHD, SI and HY participated in editing it. All authors read and approved the final manuscript.

## Supplementary Material

Additional file 1: Figure S1 Representative results of immunostaining with novel anti-CD26 mAbs. The tissue specimens of liver, kidney, prostate or two cases of malignant mesothelioma were stained with the hybridoma supernatant (clone 1, 5, 11 or 16), counterstained with hematoxylin (original magnification, 200X). **Figure S2.** Analysis of crossreactivity of novel anti-CD26 mAbs with humanized anti-CD26 mAb. Jurkat-CD26WT cells were pretreated with unlabeled humanized anti-CD26 mAb (YS110) (blue lines) or human control IgG (red lines), and then treated with the hybridoma supernatant (clone 1, 5, 11, 16, 18 or 19) or purified mouse anti-CD26 mAb (1F7 or 5F8), and subsequently stained with PE-labeled anti-mouse Ig pAb, or stained with Alexa Fluor 647-labeled YS110. Data were analyzed by flow cytometry, and the representative histograms are shown. The gray areas in each histogram show the data of isotype control. **Figure S3.** Blocking experiment of novel anti-CD26 mAb binding to CD26. Jurkat-CD26WT cells were pretreated with the hybridoma supernatant (clone 1, 5, 11, 16, 18 or 19) (blue lines) or mouse IgG_1_ isotype control (Contl. IgG) (red lines), and subsequently stained with Alexa Fluor 647-labeled anti-CD26 mAbs or PE-labeled anti-mouse Ig pAb, and analyzed by flow cytometry. The representative histograms are shown, and the gray areas in each histogram show the data of isotype control. Data shown are repeated twice with similar results. **Figure S4.** Staining for CD26 expression on COS-7 cells transfected with CD26 deletion mutants by novel anti-CD26 mAbs. cDNA of deleted CD26 was cotransfected with GFP-expressing plasmid to COS-7 cells. After 24 h, the transfected cells were stained with Alexa Fluor 647-labeled anti-CD26 mAbs or isotype control, and analyzed by flow cytometry. The representative histograms of Alexa Fluor 647 were obtained by gating for GFP positive cells among all acquired cells, and the gray areas in each histogram show the data of isotype control. Click here for file

## References

[B1] Fox DA Hussey RE Fitzgerald KA Acuto O Poole C Palley L Daley JF Schlossman SF Reinherz EL Ta1, a novel 105 KD human T cell activation antigen defined by a monoclonal antibody J Immunol 1984 133 1250 6 6205075

[B2] Nanus DM Engelstein D Gastl GA Gluck L Vidal MJ Morrison M Finstad CL Bander NH Albino AP Molecular cloning of the human kidney differentiation antigen gp160: human aminopeptidase A Proc Natl Acad Sci USA 1993 90 7069 73 10.1073/pnas.90.15.7069 8346219 PMC47077

[B3] Tanaka T Camerini D Seed B Torimoto Y Dang NH Kameoka J Dahlberg HN Schlossman SF Morimoto C Cloning and functional expression of the T cell activation antigen CD26 J Immunol 1992 149 481 6 1352530

[B4] Morimoto C Schlossman SF The structure and function of CD26 in the T-cell immune response Immunol Rev 1998 161 55 70 10.1111/j.1600-065X.1998.tb01571.x 9553764

[B5] Ohnuma K Dang NH Morimoto C Revisiting an old acquaintance: CD26 and its molecular mechanisms in T cell function Trends Immunol 2008 29 295 301 10.1016/j.it.2008.02.010 18456553

[B6] De Meester I Korom S Van Damme J Scharpe S CD26, let it cut or cut it down Immunol Today 1999 20 367 75 10.1016/S0167-5699(99)01486-3 10431157

[B7] von Bonin A Huhn J Fleischer B Dipeptidyl-peptidase IV/CD26 on T cells: analysis of an alternative T-cell activation pathway Immunol Rev 1998 161 43 53 10.1111/j.1600-065X.1998.tb01570.x 9553763

[B8] Morimoto C Torimoto Y Levinson G Rudd CE Schrieber M Dang NH Letvin NL Schlossman SF 1 F7, a novel cell surface molecule, involved in helper function of CD4 cells J Immunol 1989 143 3430 9 2479677

[B9] Masuyama J Yoshio T Suzuki K Kitagawa S Iwamoto M Kamimura T Hirata D Takeda A Kano S Minota S Characterization of the 4C8 antigen involved in transendothelial migration of CD26(hi) T cells after tight adhesion to human umbilical vein endothelial cell monolayers J Exp Med 1999 189 979 90 10.1084/jem.189.6.979 10075981 PMC2193050

[B10] Ohnuma K Inoue H Uchiyama M Yamochi T Hosono O Dang NH Morimoto C T-cell activation via CD26 and caveolin-1 in rheumatoid synovium Mod Rheumatol 2006 16 3 13 10.3109/s10165-005-0452-4 16622717 PMC2779407

[B11] Hatano R Ohnuma K Yamamoto J Dang NH Morimoto C CD26-mediated co-stimulation in human CD8(+) T cells provokes effector function via pro-inflammatory cytokine production Immunology 2013 138 165 72 10.1111/imm.12028 23113658 PMC3575769

[B12] Hatano R Ohnuma K Yamamoto J Dang NH Yamada T Morimoto C Prevention of acute graft-versus-host disease by humanized anti-CD26 monoclonal antibody Br J Haematol 2013 162 263 77 10.1111/bjh.12378 23692598

[B13] Havre PA Abe M Urasaki Y Ohnuma K Morimoto C Dang NH The role of CD26/dipeptidyl peptidase IV in cancer Front Biosci 2008 13 1634 45 10.2741/2787 17981655

[B14] Ho L Aytac U Stephens LC Ohnuma K Mills GB McKee KS Neumann C LaPushin R Cabanillas F Abbruzzese JL In vitro and in vivo antitumor effect of the anti-CD26 monoclonal antibody 1 F7 on human CD30+ anaplastic large cell T-cell lymphoma Karpas 299 Clin Cancer Res 2001 7 2031 40 11448921

[B15] Inamoto T Yamochi T Ohnuma K Iwata S Kina S Inamoto S Tachibana M Katsuoka Y Dang NH Morimoto C Anti-CD26 monoclonal antibody-mediated G1-S arrest of human renal clear cell carcinoma Caki-2 is associated with retinoblastoma substrate dephosphorylation, cyclin-dependent kinase 2 reduction, p27(kip1) enhancement, and disruption of binding to the extracellular matrix Clin Cancer Res 2006 12 3470 7 10.1158/1078-0432.CCR-06-0361 16740772

[B16] Inamoto T Yamada T Ohnuma K Kina S Takahashi N Yamochi T Inamoto S Katsuoka Y Hosono O Tanaka H Humanized anti-CD26 monoclonal antibody as a treatment for malignant mesothelioma tumors Clin Cancer Res 2007 13 4191 200 10.1158/1078-0432.CCR-07-0110 17634548

[B17] Aoe K Amatya VJ Fujimoto N Ohnuma K Hosono O Hiraki A Fujii M Yamada T Dang NH Takeshima Y Inai K Kishimoto T Morimoto C CD26 overexpression is associated with prolonged survival and enhanced chemosensitivity in malignant pleural mesothelioma Clin Cancer Res 2012 18 1447 56 10.1158/1078-0432.CCR-11-1990 22261805

[B18] Dong RP Tachibana K Hegen M Scharpe S Cho D Schlossman SF Morimoto C Correlation of the epitopes defined by anti-CD26 mAbs and CD26 function Mol Immunol 1998 35 13 21 10.1016/S0161-5890(98)80013-8 9683260

[B19] Yamada K Hayashi M Madokoro H Nishida H Du W Ohnuma K Sakamoto M Morimoto C Yamada T Nuclear localization of CD26 induced by a humanized monoclonal antibody inhibits tumor cell growth by modulating of POLR2A transcription PLoS One 2013 8 e62304 10.1371/journal.pone.0062304 23638030 PMC3639274

[B20] Takebe Y Seiki M Fujisawa J Hoy P Yokota K Arai K Yoshida M Arai N SR alpha promoter: an efficient and versatile mammalian cDNA expression system composed of the simian virus 40 early promoter and the R-U5 segment of human T-cell leukemia virus type 1 long terminal repeat Mol Cell Biol 1988 8 466 72 2827008 10.1128/mcb.8.1.466-472.1988 PMC363152

[B21] Tanaka J Miwa Y Miyoshi K Ueno A Inoue H Construction of Epstein-Barr virus-based expression vector containing mini-oriP Biochem Biophys Res Commun 1999 264 938 43 10.1006/bbrc.1999.1617 10544034

[B22] Tanaka T Duke-Cohan JS Kameoka J Yaron A Lee I Schlossman SF Morimoto C Enhancement of antigen-induced T-cell proliferation by soluble CD26/dipeptidyl peptidase IV Proc Natl Acad Sci USA 1994 91 3082 6 10.1073/pnas.91.8.3082 7909158 PMC43519

[B23] Ikushima H Munakata Y Ishii T Iwata S Terashima M Tanaka H Schlossman SF Morimoto C Internalization of CD26 by mannose 6-phosphate/insulin-like growth factor II receptor contributes to T cell activation Proc Natl Acad Sci USA 2000 97 8439 44 10.1073/pnas.97.15.8439 10900005 PMC26966

[B24] Tanaka T Kameoka J Yaron A Schlossman SF Morimoto C The costimulatory activity of the CD26 antigen requires dipeptidyl peptidase IV enzymatic activity Proc Natl Acad Sci USA 1993 90 4586 90 10.1073/pnas.90.10.4586 7685106 PMC46557

[B25] Saleh HA Jin B Barnwell J Alzohaili O Utility of immunohistochemical markers in differentiating benign from malignant follicular-derived thyroid nodules Diagn Pathol 2010 5 9 10.1186/1746-1596-5-9 20181018 PMC2831001

[B26] de Matos LL Del Giglio AB Matsubayashi CO de Lima Farah M Del Giglio A da Silva Pinhal MA Expression of CK-19, galectin-3 and HBME-1 in the differentiation of thyroid lesions: systematic review and diagnostic meta-analysis Diagn Pathol 2012 7 97 10.1186/1746-1596-7-97 22888980 PMC3523001

[B27] Hua X Yu L Huang X Liao Z Xian Q Expression and role of fibroblast activation protein-alpha in microinvasive breast carcinoma Diagn Pathol 2011 6 111 10.1186/1746-1596-6-111 22067528 PMC3228672

[B28] Lu Z Qi L Bo XJ Liu GD Wang JM Li G Expression of CD26 and CXCR4 in prostate carcinoma and its relationship with clinical parameters J Res Med Sci 2013 18 647 52 24379839 PMC3872602

[B29] Yamaguchi U Nakayama R Honda K Ichikawa H Hasegawa T Shitashige M Ono M Shoji A Sakuma T Kuwabara H Distinct gene expression-defined classes of gastrointestinal stromal tumor J Clin Oncol 2008 26 4100 8 10.1200/JCO.2007.14.2331 18757323

[B30] Torigoe T Asanuma H Nakazawa E Tamura Y Hirohashi Y Yamamoto E Kanaseki T Hasegawa T Sato N Establishment of a monoclonal anti-pan HLA class I antibody suitable for immunostaining of formalin-fixed tissue: unusually high frequency of down-regulation in breast cancer tissues Pathol Int 2012 62 303 8 10.1111/j.1440-1827.2012.02789.x 22524657

