# Supplementary figures and images for: Establishment of monoclonal anti-human CD26 antibodies suitable for immunostaining of formalin-fixed tissue
Source: Diagn Pathol. 2014 Feb 6;9:30. doi: 10.1186/1746-1596-9-30 (PMC3944398; doi:10.1186/1746-1596-9-30)

Fig. S1

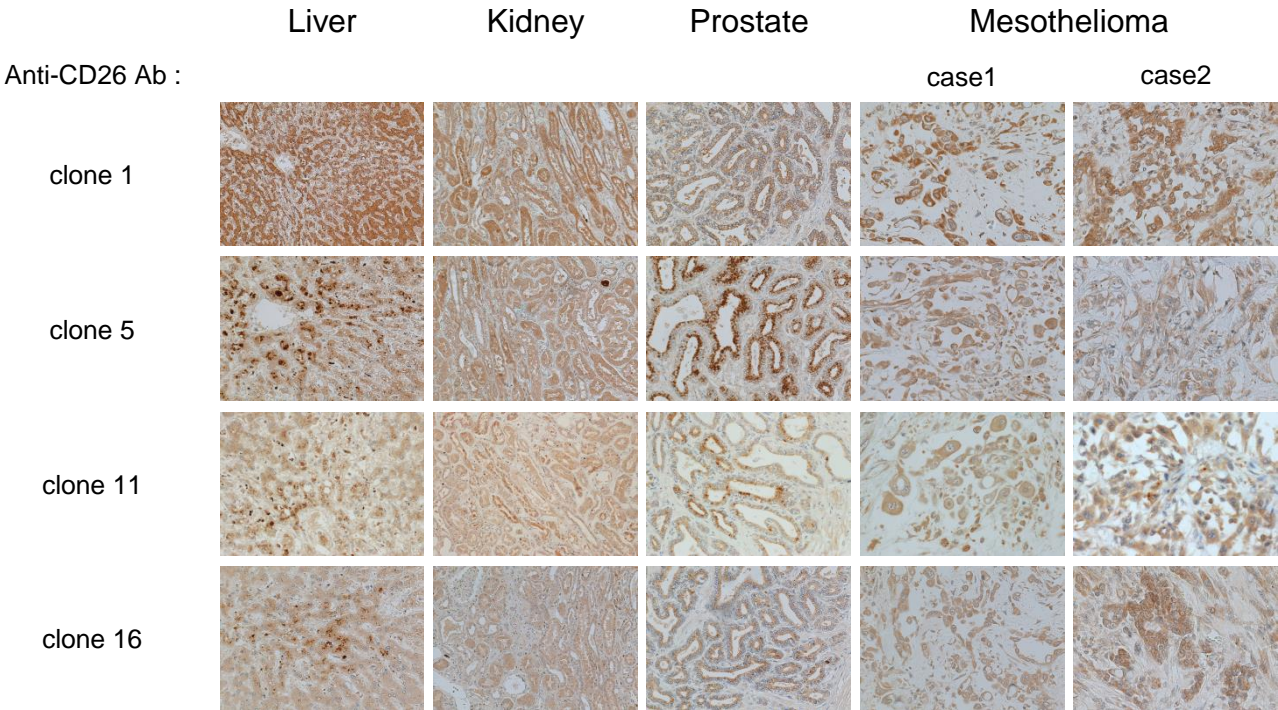

Fig. S2

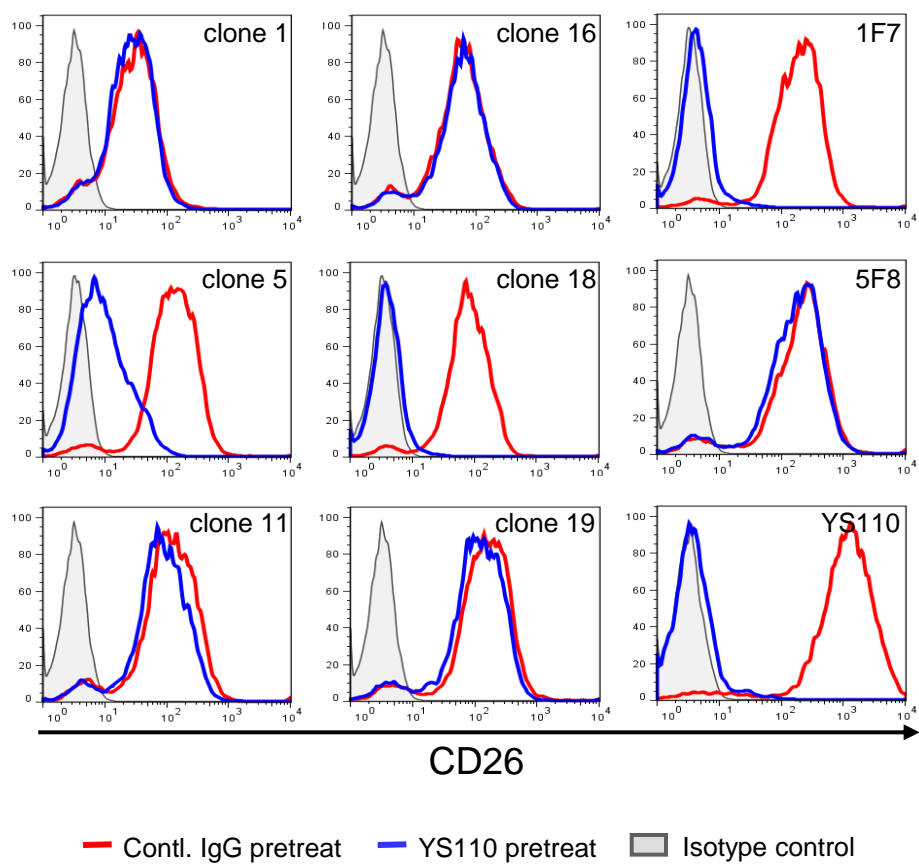

Staining :

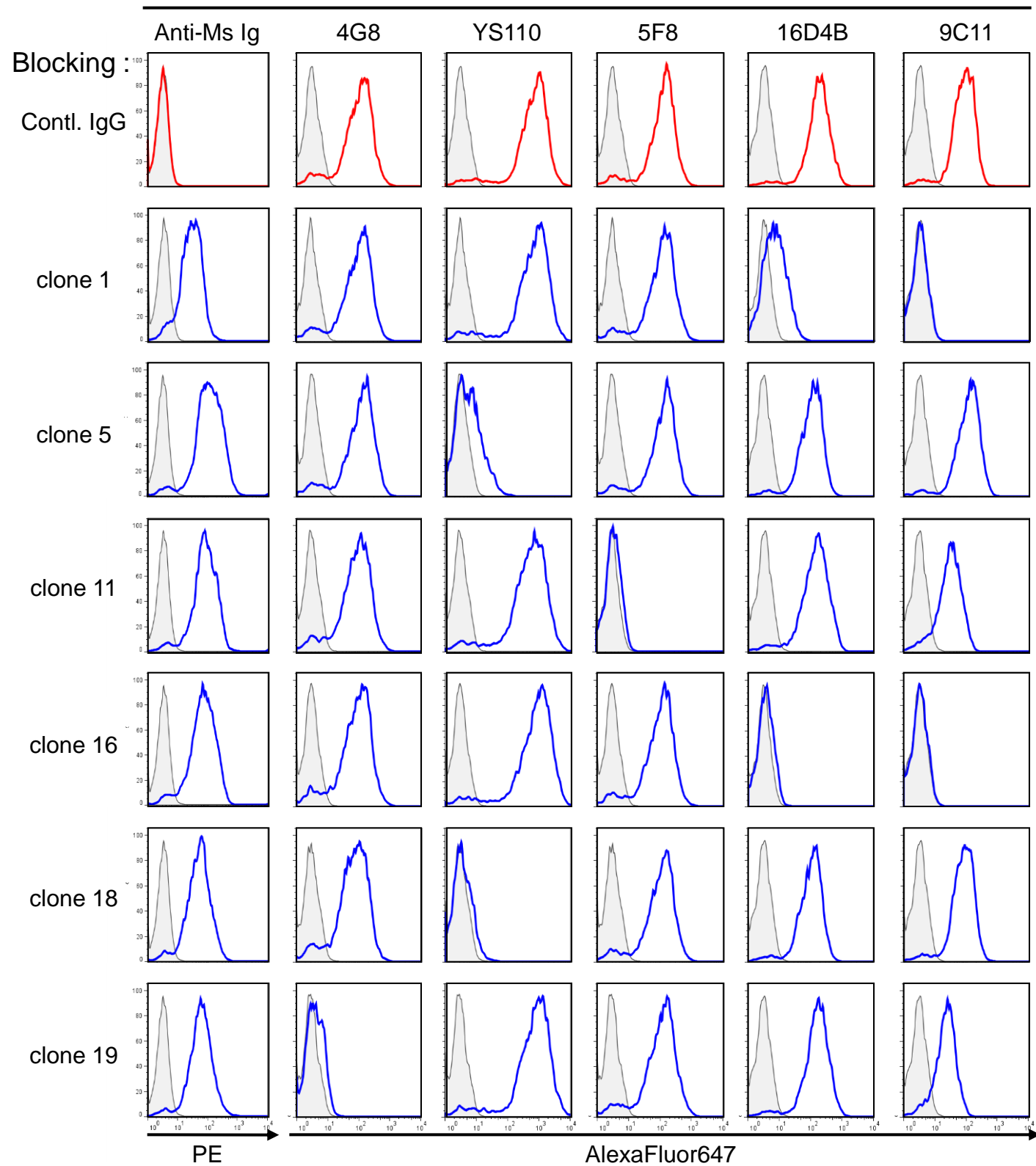

Fig. S4

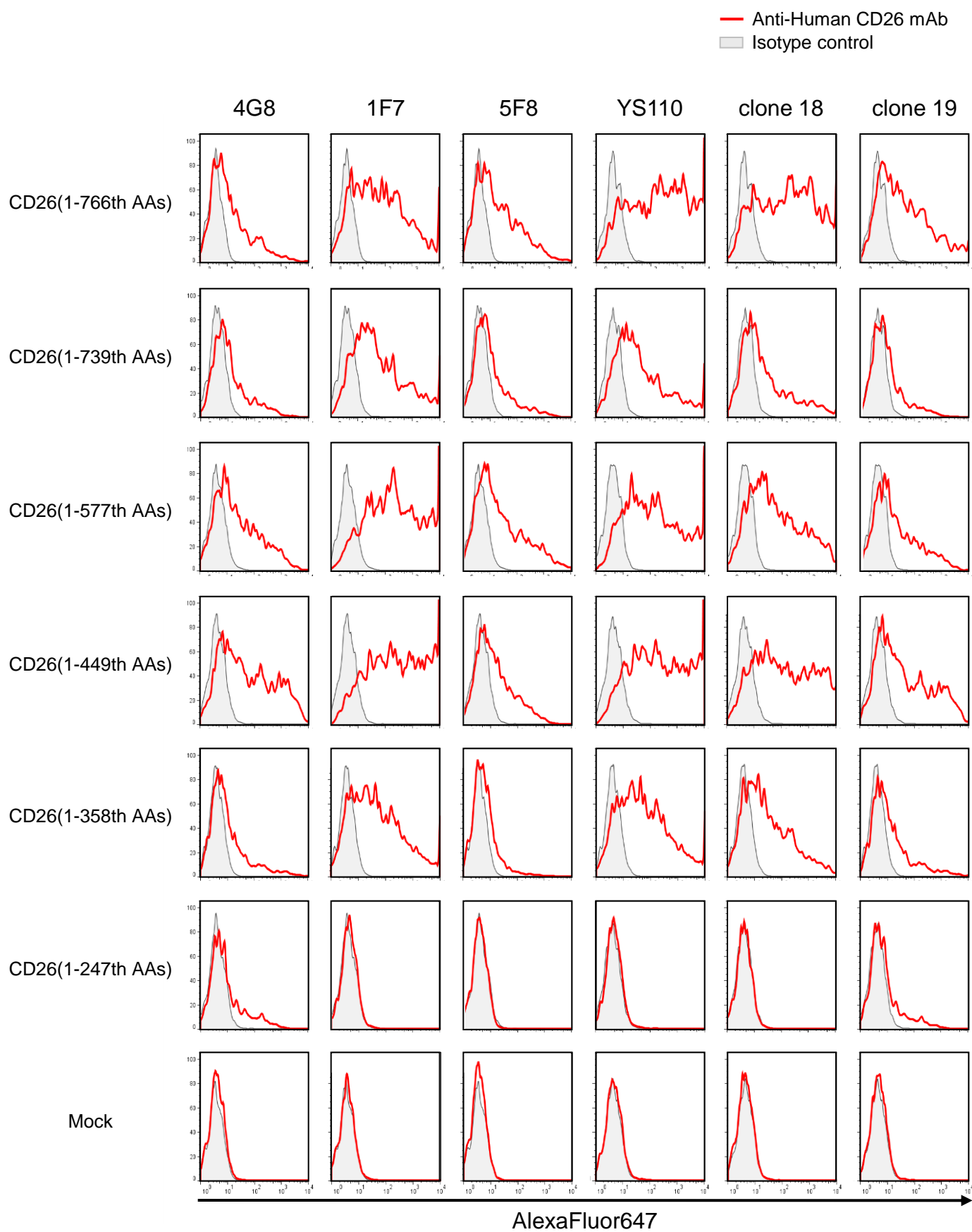

Supplement: Additional file 1: Figure S1 — Representative results of immunostaining with novel anti-CD26 mAbs. The tissue specimens of liver, kidney, prostate or two cases of malignant mesothelioma were stained with the hybridoma supernatant (clone 1, 5, 11 or 16), counterstained with hematoxylin (original magnification, 200X). Figure S2. Analysis of crossreactivity of novel anti-CD26 mAbs with humanized anti-CD26 mAb. Jurkat-CD26WT cells were pretreated with unlabeled humanized anti-CD26 mAb (YS110) (blue lines) or human control IgG (red lines), and then treated with the hybridoma supernatant (clone 1, 5, 11, 16, 18 or 19) or purified mouse anti-CD26 mAb (1F7 or 5F8), and subsequently stained with PE-labeled anti-mouse Ig pAb, or stained with Alexa Fluor 647-labeled YS110. Data were analyzed by flow cytometry, and the representative histograms are shown. The gray areas in each histogram show the data of isotype control. Figure S3. Blocking experiment of novel anti-CD26 mAb binding to CD26. Jurkat-CD26WT cells were pretreated with the hybridoma supernatant (clone 1, 5, 11, 16, 18 or 19) (blue lines) or mouse IgG1 isotype control (Contl. IgG) (red lines), and subsequently stained with Alexa Fluor 647-labeled anti-CD26 mAbs or PE-labeled anti-mouse Ig pAb, and analyzed by flow cytometry. The representative histograms are shown, and the gray areas in each histogram show the data of isotype control. Data shown are repeated twice with similar results. Figure S4. Staining for CD26 expression on COS-7 cells transfected with CD26 deletion mutants by novel anti-CD26 mAbs. cDNA of deleted CD26 was cotransfected with GFP-expressing plasmid to COS-7 cells. After 24 h, the transfected cells were stained with Alexa Fluor 647-labeled anti-CD26 mAbs or isotype control, and analyzed by flow cytometry. The representative histograms of Alexa Fluor 647 were obtained by gating for GFP positive cells among all acquired cells, and the gray areas in each histogram show the data of isotype control. [file 1746-1596-9-30-S1.pdf]
